# Construction and regulation of high active sites in montmorillonite composite catalyst for the removal of ofloxacin via persulfate activation

**DOI:** 10.1016/j.heliyon.2024.e29896

**Published:** 2024-04-22

**Authors:** Fu-zhi Huang, Ya-qi Wang, Wan-yin Gao, Xiao-qiang Cao, Yang Zhang, Ya-nan Shang, Yi-zhen Zhang, Yu-jiao Kan

**Affiliations:** a College of Safety and Environmental Engineering, Shandong University of Science and Technology, Qingdao, 266590, China; b Institute of Yellow River Delta Earth Surface Processes and Ecological Integrity, Shandong University of Science and Technology, Qingdao, 266590, China

**Keywords:** Montmorillonite composite catalyst, Active sites, Permonosulfate, Ofloxacin

## Abstract

In this study, ionic liquids (ILs) were used as organic modifiers by introducing montmorillonite nanolayers containing potential C and N active sites between the montmorillonite nanolayers. Organically modified montmorillonite (ILs-Mt-p) was further prepared by high-temperature pyrolysis under N_2_ and used for the removal of ofloxacin (OFL) by activated peroxymonosulfate (PMS). Combined with XPS and other characterization analyses, it was found that the catalyst materials prepared from different organic modifiers had similar surface functional groups and graphitized structures, but contained differences in the types and numbers of C and N active sites. The catalyst (3CPC-Mt-p) obtained after pyrolysis of montmorillonite modified with cetylpyridinium chloride (CPC) had optimal catalytic performance, in which graphitic C, graphitic N, and carbonyl group (C=O) could synergistically promote the activation of PMS by electron transfer, and 77.3 % of OFL could be removed within 60 min. The effects of OFL concentration, initial pH, and anions on the effects of OFL removal by the 3CPC-Mt-p/PMS system were further investigated. Satisfactory degradation results were obtained over a wide pH range. Cl^−^ promoted the system to degrade OFL, while the presence of SO_4_^2−^, H_2_PO_4_^−^ and HA showed some inhibition, but overall the 3CPC-Mt-p catalysts had a strong anti-interference ability, showing good application prospects. The quenching experiments and EPR tests showed that O_2_^−-^ and ^1^O_2_ in the 3CPC-Mt-p/PMS system were the main reactive oxygen species for the degradation of OFL, and •OH was also involved in the reaction. This study provides ideas for the construction and modulation of active sites in mineral materials such as montmorillonite and broadens the application of montmorillonite composite catalysts in advanced oxidation processes for the treatment of antibiotic wastewater.

## 1 Introduction

The water pollution caused by the misuse of antibiotics has become a global problem, endangering aquatic ecosystems and human health by inducing antibiotic resistance and endocrine disruption. Residual antibiotics enter the ecosystem through various pathways, with the highest detected concentration in surface water reaching levels of μg/L [1]. There are several water treatment technologies that can be used to eliminate antibiotic contamination in water. These treatment technologies can be divided into physical technologies (adsorption, membrane filtration, etc.), chemical technologies (UV irradiation, photocatalysis, microwave catalysis, etc.) and biological technologies (activated sludge method, biofilm method, enzyme degradation etc.) [[2], [3], [4], [5]]. Among these technologies, advanced oxidation processes (AOPs) gradually draw more attention for their fast reaction rates, versatility and flexibility and non-secondary pollution [6,7]. Heterogeneous advanced oxidation processes based on persulfate (PS-AOPs) is an emerging water treatment technology for antibiotic contamination, due to their high oxidation efficiency and environmental friendliness, becoming a research hotspot in recent years [8,9]. Compared with peroxodisulfate (PDS), peroxymonosulfate (PMS) is more easily activated due to its asymmetric molecular structure and shorter O–O bond length, which has been applied by many scholars in the field of environmental remediation. PMS can be activated in various ways, including ultrasound treatment [10], UV irradiation [11], electrochemical oxidation [12], and catalytic materials (transition metal ions or metal oxides [[13], [14], [15]], non-metallic carbon materials [16,17], and mineral catalytic materials [[18], [19], [20], [21]]). However, the high energy cost limits the application of activation methods such as ultrasound, UV irradiation, and electrochemical oxidation. Metal ions in metal-based catalysts are prone to leaching, while carbon-based materials have low catalytic activity and are difficult to separate and recover in practical applications. Mineral materials, on the other hand, are emerging as new types of catalyst materials due to their low cost and effective activation of PMS [22,23].

Montmorillonite, as one of the low-cost and environmentally friendly mineral materials, has been widely used in the treatment of antibiotic wastewater. However, natural montmorillonite has low catalytic activity, and introducing new active sites by loading other active components can improve its catalytic activity. At present, most studies focus on introducing metal active sites into montmorillonite gradients. Wu et al. [24] prepared composite materials (nZVI/OMt) by loading nZVI on organically modified montmorillonite (OMt), and used it to activate PDS to degrade sulfamethoxazole (SMZ), achieving a degradation of over 97 % of 20 mg/L SMZ in 10 min. Xiao et al. [25] successfully prepared Fe/C–Mt composite catalyst and used it to activate PMS to remove OFL with an efficiency of 96.2 %. Peng et al. [26] successfully prepared Fe_3_O_4_/MMT composite material with a simple method and used it for the activation and degradation of enrofloxacin (ENR) by PS. Through the synergistic effect of catalyst adsorption and degradation, more than 90 % of ENR could be removed within 60 min. However, research on introducing non-metallic active sites in montmorillonite with non-metallic activity is relatively limited. The excellent expansion ability and high cation exchange ability of montmorillonite can be used to carry out organic modification by exchanging cations between montmorillonite layers with ionic liquids, thereby introducing potential C and N active sites into the interlayer. Non-metallic active sites have lower biological toxicity, which can fundamentally avoid the problem of metal ion leakage. Besides, it is reported that the type and quantity of active sites in the catalyst are closely related to the pathway by which the catalyst activates PMS to degrade pollutants. For example, active sites of graphite C and graphite N mainly correspond to non-radical, which could enhance the degradation performance for organic pollutants [27]. As far as we know, it is not been fully invested to construct non-metallic active sites in montmorillonite composite through organically modified montmorillonite for PMS activation.

This study aims to construct and regulate C and N active sites within the interlayers of montmorillonite through organic modification. Cetylpyridinium chloride (CPC) and 1-hexadecyl-3-methylimidazolium chloride (C16mimCl) are two typical and frequently-used organic modifiers with the same chain lengths and different head groups, which may affect their combination with montmorillonite. Herein, CPC and C16mimCl were selected and loaded onto the interlayers and surfaces of montmorillonite. The modifiers were then thermally treated at high temperatures to create C and N active sites within the montmorillonite interlayers, resulting in a composite catalyst with enhanced capability to catalytically active PMS for organic pollution. Ofloxacin (OFL), a widely used typical antibiotic, was selected as a targeted pollutant. The types and quantities of C and N active sites were controlled by adjusting the type and content of the ionic liquids. The excellent performance of the catalyst was determined through the characterization of its crystal structure, specific surface area, elemental content, and oxidation states. The study also investigated the degradation characteristics of OFL under different influencing factors. Furthermore, the degradation mechanism was explored through radical quenching experiments and electron paramagnetic resonance (EPR) tests. Characterization of the catalyst before and after use confirms the dominant mechanism of the reaction process and the related mechanisms of catalyst deactivation. This research will contribute to a better understanding of the role of active sites in catalysts in activating PMS for degrading pollutants, and it is particularly important for utilizing clay material as a value-added product in future developments.

## 2 Experimental materials and methods

### 2.1 Reagents

Details of the reagents used in this work are described in Text S1 of the Supporting Information.

### 2.2 Sample preparation

Two typical ionic liquids of cetylpyridinium chloride (CPC) and 1-hexadecyl -3-methylimidazolium chloride (C_16_mimCl) were selected as organic modifiers for preparing organically modified montmorillonite catalysts. The cation exchange capacity (CEC) is a key parameter to describe the ability of montmorillonite to adsorb and release cations, related to its negative charge, the hydration, expansion, and dispersion capacity. In a typical synthesis procedure, 1 g of montmorillonite, with CEC of 82 mmol/100 g, and a serious number of organic modifiers based on the CEC of montmorillonite were employed for preparation. The prepared samples were noted as nCPC-Mt and nC_16_mimCl-Mt, as shown in Tables S1 and S2. The organic modifiers were added into 50 mL of deionized water in a conical flask (150 mL), which was then sonicated for 5 min for complete dissolution. The organic modifier solution was mixed with a certain amount of pre-prepared Na–Mt, which was then placed in a constant-temperature water bath shaker (40 °C) and shaken for 5 h. The obtained composite was separated by centrifuging at 5000 rpm for 7 min. The sample was washed with deionized water and centrifuged for 6 cycles and dried in an oven at 80 °C for 24 h. Then the dried sample was grounded and sieved at 200-mesh to obtain ionic liquids modified montmorillonite (ILs-Mt). The precursor of ILs-Mt prepared above was pyrolyzed into a tube furnace at 700 °C under N_2_ atmosphere, with a heating rate of 5 °C min^−1^. After cooling, the carbonized catalyst was preserved in a brown sample bottle to obtain organically modified montmorillonite (ILs-Mt-p), which were noted as nCPC-Mt-p and nC_16_mimCl-Mt-p.

### 2.3 Experimental methods and analytical methods

The OFL degradation experiment was conducted in a 150 mL conical flask containing 50 mL OFL solution, a certain amount of PMS, and a catalyst. The conical flask was placed in a constant-temperature oscillator at 140 rpm. The initial pH of the solution was adjusted by 0.1 M NaOH and H_2_SO_4_. At proper sampling time, 0.5 mL of the water samples were filtered with a 0.22 μM filter and 1 mL methanol was added to terminate the degradation reaction. The reactive oxygen species (ROS) generated in the solution were eliminated with corresponding quenchers (Table S3) to examine their contribution to OFL degradation. The concentration of OFL was determined by a high-performance liquid chromatograph (HPLC) equipped with a C-18 column. All experiments were performed twice in parallel to ensure reproducibility. Details of experimental methods and analytical methods are stated in Text S2.

### 2.4 Sample characterization methods and DFT calculation

Details of the characterization methods including X-ray diffractometer (XRD), fourier-transform infrared spectrometer (FTIR), scanning electron microscope (SEM), energy dispersive spectroscopy (EDS), transmission electron microscope (TEM), analysis of specific surface area, pore size and pore volume, gas chromatograph-mass spectrometer (GC-MS), thermogravimetric analyzer (TGA), X-ray photoelectron spectroscopy (XPS), electron paramagnetic resonance (EPR), etc. are described in Text S3. The Fukui function of OFL were derived from DFT calculation. The visualization of Fukui isosurfaces were conducted by Multiwfn 3.8 [28] and VMD 1.9.3 software [29].

## 3 Results and discussion

### 3.1 Characterization of the samples

**Fig. 1:**
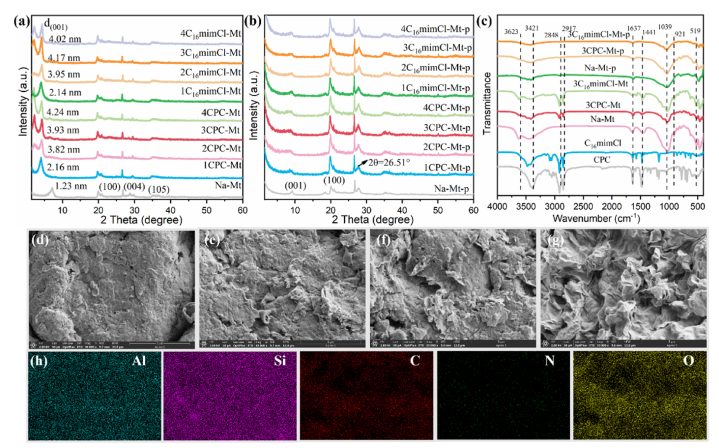
XRD patterns of ILs-Mt before (a) and after (b) high-temperature pyrolysis, (c) FTIR spectra of different samples, SEM images of different samples and the distribution of each element in 3CPC-Mt-p (SEM: (d) Na–Mt, (e) 1CPC-Mt, (f) 3CPC-Mt, (g) 3CPC-Mt-p; (h) 3CPC-Mt-p Mapping of Al, Si, C, N, O).

The interlayer spacing of montmorillonite is an important feature that reflects its structure. Under high-temperature conditions, the crystal structure of the sample will change. The XRD characterization results of ILs-Mt before and after pyrolysis (Fig. 1a and b) show that the diffraction peak shifts to a lower angle, which suggests the d_001_ value of ILs-Mt significantly increases compared with unmodified montmorillonite. This result also indicates that CPC and C_16_mimCl modifiers have entered the interlayer or surface of montmorillonite, significantly expanding the interlayer spacing. It can be observed that the two modifiers have little influence on the interlayer spacing of montmorillonite [30]. As the amount of CPC and C_16_mimCl increases to the amount of double CEC of montmorillonite, the d_001_ diffraction peak splits into two peaks. When the amount of modifier was increased to triple CEC, the spacing between montmorillonite layers no longer changed significantly. This is because the adsorbed organic matter between the layers is gradually saturated. After pyrolysis, the d_001_ values of all samples decreased significantly, which was caused by the removal of interlayer water [31]. ILs-Mt-p still retains the characteristic diffraction peaks of montmorillonite, which indicates that high-temperature pyrolysis could not destroy the pristine structure of montmorillonite. The diffraction peak of the graphitic carbon phase crystal appears at 2θ = 26.51°. This indicates that the ionic liquid formed a graphitic carbon structure after high-temperature pyrolysis. This result is consistent with the research results of Yang et al. [32].

FTIR was used to reveal the changes in functional groups and structure of the modified catalytic material before and after pyrolysis, which is present in Fig. 1c. Typical montmorillonite absorption peaks (519 cm^−1^, 921 cm^−1^, and 1039 cm^−1^) are observed in various samples, indicating that the loaded organic components and pyrolysis did not disrupt the structure of montmorillonite. The characteristic peaks of CPC and C_16_mimCl appear in the ILs-Mt-p spectrum. This suggests that ILs had been successfully loaded on montmorillonite. The three characteristic bands at 1441, 2848, and 2917 cm^−1^ can be assigned to the –CH_2_ asymmetric stretching vibration absorption peak, C–H symmetric stretching vibration peak, and -(CH_2_)n-asymmetric stretching vibration peak. Moreover, the intensity of the characteristic peak of •OH of adsorbing water at 1637 cm^−1^ becomes weak after organic modification. This indicates that the loading of ILs has a hydrophobic effect [33]. The absorption peaks of CPC and C_16_mimCl at 3421 cm^−1^ are attributed to the bending vibration of -N-H. This is due to the strong interaction between the N–H group in the modifier and Mt, resulting in its stretching vibration. After high-temperature pyrolysis, the intensity of the absorption peak in ILs-Mt-p significantly weakened. The –OH characteristic peaks related to adsorbed water and combined water between the montmorillonite layers at 1637 cm^−1^ and 3623 cm^−1^ essentially disappeared. This result may be attributed to the complete dehydration of montmorillonite under high-temperature conditions.

The morphology and element distribution of the samples were characterized by SEM (30,000× magnification) and EDS, as are shown in Fig. 1d-l, Fig. S1, and Fig. S2. Na–Mt has a fish scale-like lamellar structure with a compact and compact surface (Fig. 1d). There is no obvious difference in the morphology of the modified montmorillonite obtained by using the two modifiers at the same dosage (Fig. 1e and f *vs.* Figs. S1b and c). It has a relatively loose layered structure. As the amount of modifier increases, the surface of montmorillonite becomes looser, and the lamellar structure is out of shape of a curled fishscale. This is due to the insertion of ILs expanding the spacing between montmorillonite layers. After high-temperature pyrolysis, the surface of ILs-Mt-p becomes wrinkled (Fig. 1f,g & Figs. S1c and d). This is attributed to the polycondensation reaction of organic matter pyrolyzed by high temperature, transforming it into sheet-like carbon materials [19]. From the Mapping diagram (Fig. 1h), it can be seen that Al, Si, C, N, and O elements are evenly dispersed on the surface of 3CPC-Mt-p, confirming that the organic modifier is evenly distributed. To further verify the catalyst structure, the samples were characterized by TEM (Fig. S3). It can be seen that Na-Mt-p exhibits a smooth lamellar structure, while 3CPC-Mt-p and 3C_16_mimCl-Mt-p exhibit transparent wrinkled layers. This is attributed to the conversion of organic matter into carbon materials during the pyrolysis process, which is consistent with the SEM characterization results.

Further, a thermogravimetric-gas chromatography-mass spectrometry (TG-GC-MS) coupled platform was used for the analysis of various gas phase products produced during the pyrolysis process of ILs-Mt (Fig. S4). At 30–310 °C, the corresponding peak with a retention time of 3.42 ± 0.1 min is the olefin fragment ion C_3_H_5_^+^ (*m*/*z* = 41) peak. Pyridine nitrogen (*m*/*z* = 79) is shown in the TIC diagrams at the sampling temperature points of 310 °C and 700 °C. As the temperature increases, the pyridine nitrogen content increases, which indicates that more CPC in the modified montmorillonite undergoes pyrolysis. As CPC-Mt were pyrolyzed at the deep stage (310–700 °C), the main newly generated gaseous products are the olefin fragment ion C_16_H_32_^+^ (*m*/*z* = 55) and the linear alkyl fragment ion C_4_H_9_^+^ (*m*/*z* = 57). This may be attributed to the fact that the undecomposed alkyl chain begins to break before 310 °C. The pattern of gas phase products during the pyrolysis process of C_16_mimCl-Mt is similar to that of CPC-Mt. The corresponding substance peak at 30–310 °C is methyl chloride (*m*/*z* = 50). At the deep pyrolysis stage (310–700 °C), the main gaseous product is the olefin fragment ion C_3_H_5_^+^ (*m*/*z* = 41), C_16_H_32_^+^ (*m*/*z* = 55) and 1-methylimidazole (*m*/*z* = 82). These results show that different modifiers supported on montmorillonite could produce different structures and functional group types after pyrolysis, thus showing different catalytic properties.

**Fig. 2:**
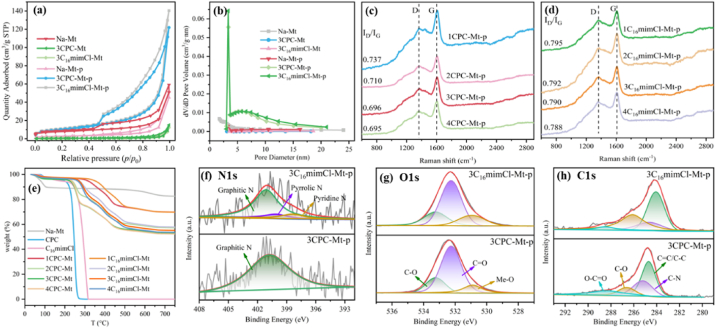
Comparison of N_2_ adsorption-desorption isotherms (a) and pore size distribution (b), Raman spectra of different samples(c) and(d), TGA plots of different samples (e), high-resolution N 1s (g), O 1s (e) and C 1s (h) peak split spectra of different samples.

The specific surface area and pore structure of the samples before and after high-temperature pyrolysis were analyzed through a nitrogen adsorption-desorption experiment, as is shown in Fig. 2a–b. All adsorption-desorption isotherms exhibit standard type IV which indicates that the material has mesoporous characteristics [4,34]. In addition, the H_3_ type hysteresis loop exists between the adsorption and desorption curves in the range of *p/p*_0_ from 0.45 to 0.98, indicating that there is a narrow slit-like pore structure formed by the accumulation of fine particles in the sample [35]. The N_2_ adsorption capacity of both 3CPC-Mt and 3C_16_mimCl-Mt decreased, which is due to the successful introduction of organic modifiers into montmorillonite occupying its adsorption sites. The adsorption capacity of the sample increased significantly after pyrolysis which is attributed to the high-temperature pyrolysis of ILs between the montmorillonite layers, forming a graphitic carbon structure with a larger specific surface area. From Fig. 2b and Table S4, it can be seen that the pore sizes of each sample are mainly distributed between 2 and 15 nm, which suggests that the catalyst mainly has a mesoporous structure [36,37]. After modification, the pore volume of the sample became smaller, while the average pore diameter increased, which may be due to ILs occupying the interlayer pores of montmorillonite through the tail–tail interactions [38]. In addition, the steric hindrance and electrostatic repulsion between molecules would open the sample pores and increase the average pore diameter [39]. Combined with Table S4, it can be seen that high-temperature pyrolysis causes the collapse of the montmorillonite lamellar structure. After pyrolysis of organically modified montmorillonite, the specific surface area and pore volume increased, while the average pore diameter decreased. This may be caused by the carbonization of ILs during high-temperature pyrolysis, forming rich graphitic carbon and pore structures.

Raman spectra can be used to reflect the degree of defects or graphitization of the sample through the I_D_/I_G_ value. The lower the value of I_D_/I_G_, the higher the degree of graphitization of the material and vice versa [40]. It can be seen from Fig. 2c–d that as the organic matter loading increases, the I_D_/I_G_ values of the two modified montmorillonites gradually decrease, which indicates that the increase in organic matter could increase the degree of graphitization of the catalyst. With the same addition amount, the I_D_/I_G_ value of CPC-Mt-p is lower than that of C_16_mimCl-Mt-p, which shows that the carbon material formed after CPC pyrolysis has a higher degree of graphitization. Subsequent experiments showed that the catalytic performance of CPC-Mt-p was significantly better than that of C_16_mimCl-Mt-p. It was speculated that the graphitized structure of the sample may be beneficial to the activation of PMS for the degradation of OFL.

TGA was used to analyze the thermal stability of the sample. As shown in Fig. 2e, Na–Mt exhibits weight loss at around 80 °C, which is attributed to the evaporation of water in the sample [41]. The weight loss rate of modified montmorillonite decreases, indicating that the alkyl chain of ILs exchanges the hydrated inorganic cations between the montmorillonite layers. As a result, the interlayer water decreases [42], which is consistent with the FTIR characterization results. Compared with pure CPC and C_16_minCl, the thermal stability of CPC and C_16_minCl in montmorillonite is significantly improved, which shows that montmorillonite has a shielding effect on the thermal decomposition of CPC and C_16_minCl [43]. When the temperature exceeds 500 °C, the quality of all modified montmorillonites tends to be stable. The weight loss rate of organically modified montmorillonites increases with the increase in the amount of modifier. On the other hand, when the dosage of two modifiers is at the same level, the weight loss rate of CPC-Mt is slightly higher than that of C_16_minCl-Mt. This is because the adsorption capacity of CPC on montmorillonite exceeds that of C_16_minCl. Combined with the Raman spectrum test, it was shown that saturation was reached when the amount of organic matter was 3 CEC. Considering the energy consumption in material preparation, 3 CEC was selected as the optimal addition amount of organic matter in subsequent experiments.

XPS spectra can reflect the chemical composition and morphology of the material surface. As is shown in Fig. S5, N 1s, O 1s, and C 1s characteristic peaks, near 400.9 eV, 532.5 eV, and 285.1 eV respectively, can be detected in 3CPC-Mt-p and 3C_16_mimCl-Mt-p catalysts. The presence of N and C further confirms the successful loading of organic modifiers onto montmorillonite. Previous literature has shown that N doping can change the electron distribution of the carbon structure and further regulate the electronic configuration of adjacent carbon structures, which causes distortion of the carbon structure and makes it the catalyst easier to activate PMS [44,45]. From the high-resolution N 1s spectrum (Fig. 2f), it can be seen that only graphite N (401.2 eV) species exist in the 3CPC-Mt-p material, while 3C_16_mimCl-Mt-p also contains pyrrole N (400.1 eV) and pyridine N (398.4 eV) in addition to graphite N. As the content of graphite N and pyridine N in the catalyst increases, its catalytic performance can be improved. This is because graphite N is related to electron density, and pyridine N can increase the oxidation potential of the sample [46]. In Table S6, it can be seen that since the adsorption amount of CPC on montmorillonite is more than C_16_minCl, the N atom doping amount of the montmorillonite composite prepared by CPC modification is higher than that of C_16_mimCl. Therefore, it is speculated that 3CPC-Mt-p should have higher catalytic activity. From the O 1s spectrum (Fig. 2g), it can be seen that oxygen has three different chemical states. The characteristic peaks at 533.1 eV and 532.2 eV can be attributed to C=O and C–O respectively. The characteristic peak at 530.9 eV can be attributed to lattice oxygen [47,48]. It is calculated from the spectra that the corresponding ratios of C=O and C–O (A_C_=_O_/A_C-O_) of 3CPC-Mt-p and 3C_16_mimCl-Mt-p are 3.47 and 1.40 respectively, as a result that the increase in the proportion of C=O groups can improve catalytic performance of the catalyst. There is a negligible difference in the C 1s spectra (Fig. 2h) of 3CPC-Mt-p and 3C_16_mimCl-Mt-p [49]. It can be seen from Table S5 that the C atom content of CPC-modified montmorillonite is higher than that of C_16_mimCl with the same dosage of organic modifier. This is attributed to the fact that the adsorption amount of CPC on montmorillonite is more than C_16_minCl, which is consistent with the characterization results of TGA.

### 3.2 Catalytic properties of organically modified montmorillonite samples

**Fig. 3:**
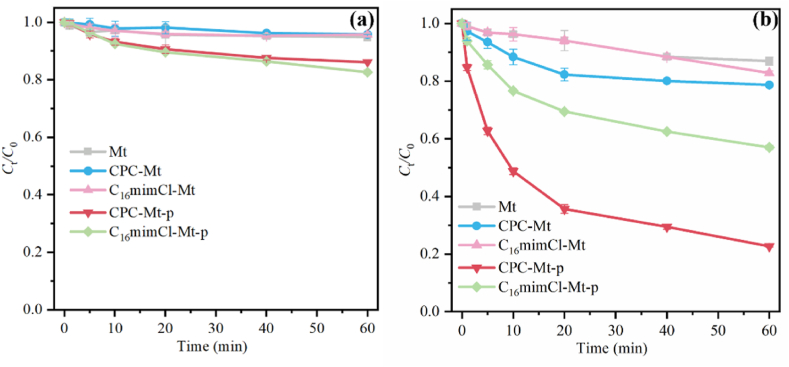
Adsorption performance of different samples on OFL (a) and degradation performance of different samples for OFL (b). (Reaction condition: catalyst dosage = 0.4 g L^−1^, [PMS] = 4 mM, [OFL] = 20 mg/L, pH = 6.8).

OFL was selected as the target pollutant to evaluate the catalytic performance of organically modified montmorillonite catalysts to activate PMS, as shown in Fig. 3a and Fig. S6a. The adsorption properties of organic-modified montmorillonite for OFL are negligible. PMS was added to study the catalytic activity of the samples. The catalytic ability of ILs-Mt without pyrolysis is limited because there are fewer active sites on its surface. After high-temperature pyrolysis, the degradation rates of OFL by 3CPC-Mt-p and 3C_16_mimCl-Mt-p increased to 77.3 % and 42.9 % respectively. This is attributed to the high-temperature pyrolysis causing the organic matter to form a graphitic carbon structure between the montmorillonite layers, so that the sample has abundant C and N active sites for PMS activation. Besides, the increase in specific surface area can increase the mass transfer rate of pollutants and PMS in the catalyst, which can also improve the catalytic performance of catalysts. Through comparison, it was found that the catalytic degradation performance of 3CPC-Mt-p was significantly better than that of 3C_16_mimCl-Mt-p, which may be attributed to the higher degree of graphitization and C=O ratio of 3CPC-Mt-p. The electron transfer ability of 3CPC-Mt-p was enhanced and the activation of PMS was promoted [49,50]. Therefore, 3CPC-Mt-p was selected as the ideal catalyst for subsequent study. In addition, it is worth noting that the performance of 3CPC-Mt-p, with a *k*_obs_ of 0.0267 min^−1^ for OFL degradation, is competitive among the reported catalysts (Table S7).

### 3.3 Effects of different factors on the degradation of ofloxacin over the 3CPC-Mt-p catalyst

**Fig. 4:**
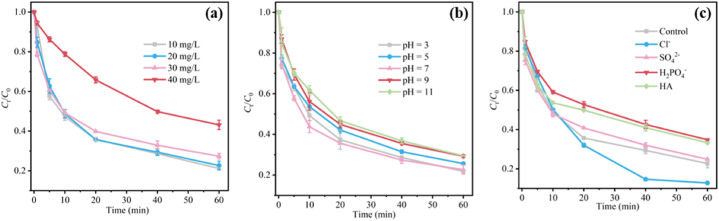
The influence of pollutant concentration on the degradation performance of OFL (a); The effect of initial pH on the degradation performance of OFL (b); Effect of anions and HA in water on the degradation performance of OFL (c).

As the target pollutant in the system, the concentration of OFL has a great influence on the degradation effect. It can be seen from Fig. 4a, the degradation efficiencies of OFL initial concentrations of 10 mg/L and 20 mg/L are approximately the same, and the *k*_obs_ are 0.0305 and 0.0267 min^−1^ respectively. When the initial concentration of OFL increased from 10 to 40 mg/L, the OFL degradation efficiency of 3CPC-Mt-p decreased from 77.3 % to 56.8 % in 60 min, and the reaction rate constant dropped significantly to 0.0148 min^−1^. This is because when the initial OFL concentration is lower than 20 mg/L, the adsorption capacity and degradation ability of the catalyst do not reach the limit. Therefore, as the initial concentration of OFL increases, the degradation efficiency does not change much. However, when the initial concentration is 40 mg/L, the degradation efficiency of OFL decreases significantly, which is caused by insufficiently activated sites of the catalyst.

The initial pH value of the solution is a key factor affecting the catalytic performance (Fig. 4b). When the pH of the solution is 7.0, the degradation efficiency is the highest (78.2 %). When the solution pH is 3.0, the system degradation efficiency can still reach 77.3 %. When the solution pH is 11.0, the OFL degradation efficiency of the system is reduced to 70.6 %. Combined with the Zeta potential of 3CPC-Mt-p (Fig. S8), in the pH range of 3.0–11.0, the surface of 3CPC-Mt-p is negatively charged. As the pH value increases, the surface of 3CPC-Mt-p Zeta potential decreases. Previous studies have shown that the pK_a1_ and pK_a2_ of PMS are 0 and 9.442 respectively [51]. Therefore, when the pH is less than 9, the main form of PMS is HSO_5_^−^, which is more easily activated by 3CPC-Mt-p, leading to the rapid degradation of OFL. Under strong alkaline conditions, the negative charge of 3CPC-Mt-p is larger, which is not conducive to SO_5_^2−^ entering the montmorillonite layer. Besides, SO_4_^•−^ will react with •OH in the solution to produce HSO_5_^−^ [52], resulting in a reduced degradation rate of OFL under alkaline conditions. Combined with the zeta potential results, the synergistic effect of PMS, OFL and 3CPC-Mt-p shows that the 3CPC-Mt-p/PMS system has high catalytic activity and practical application potential under pH = 3.0–11.0.

Normally, inorganic anions (Cl^−^, SO_4_^−^, etc) and natural organic matter (HA) in natural water environments can act as free radical scavengers to inhibit their oxidation process [53,54]. As shown in Fig. 4c, the addition of 50 mM Cl^−^ increased the degradation efficiency of OFL in this system from 77.3 % (*k*_obs_ = 0.0267 min^−1^) to 87.2 % (0.0289 min^−1^). This may be attributed to the fact that Cl^−^ can react with free radicals in the solution in which new and more active chlorine species form, such as active chlorine radicals (Cl•) [53,55]. These species are more selective for pollutants and typically tend to attack electron-rich contaminants [55,56]. This characteristic is beneficial to the degradation of OFL in the system. SO_4_^2−^ has little effect on OFL degradation, while the addition of H_2_PO_4_^−^ shows an inhibitory effect on OFL degradation. This is attributed to the fact that H_2_PO_4_^−^ has a strong affinity for the active site of the catalyst. It competes with PMS for the active site, resulting in a decrease in activation efficiency [57]. In addition, H_2_PO_4_^−^ can also interact with the hydroxyl groups on the catalyst surface, further inhibiting the adsorption and activation of PMS on the catalyst surface. The adsorption competition between HA and PMS is not conducive to the degradation of target pollutants in water [53,54]. Additionally, the adsorption and oxidation competition between HA and target pollutants further hinders the degradation process. However, when 50 mM HA is added, the degradation rate of OFL can still reach 66.7 %, which shows that the 3CPC-Mt-p/PMS system has the potential for practical applications in wastewater treatment.

### 3.4 ROS and degradation mechanism

**Fig. 5:**
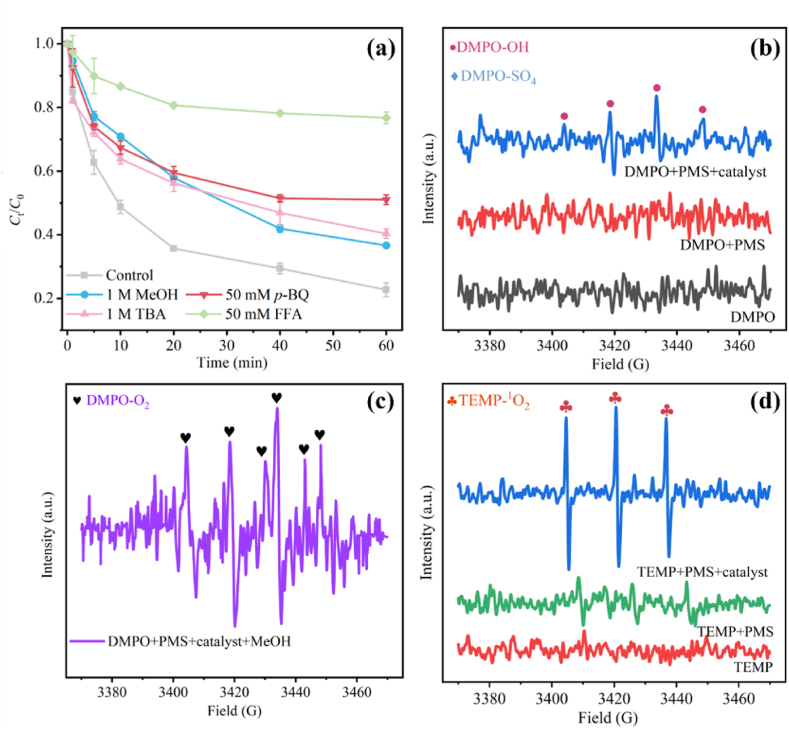
Effect of quenchers on OFL degradation (a); The EPR spectra of DMPO-SO_4_^•−^ and DMPO-OH (b), DMPO-O_2_^•−^ (c) and TEMP-^1^O_2_ (d) over 3CPC-Mt-p.

To better understand the activation mechanism, quenching experiments were performed to determine ROS in solution. MeOH is generally considered to be an effective scavenger of SO_4_^•−^ and •OH, while TBA is only effective for •OH, and *p*-BQ and FFA are used as O_2_^•−^ and ^1^O_2_ quenchers to explore their contributions. As shown in Fig. 5a, after adding MeOH and TBA, the degradation efficiency dropped to 63.4 % (*k*_obs_ = 0.0144 min^−1^) and 60.0 % (*k*_obs_ = 0.0128 min^−1^) respectively, which indicated that the presence of SO_4_^•−^ and •OH in 3CPC-Mt-p/PMS system. It is worth noting that the inhibitory effect of TBA on the reaction is greater than that of MeOH. This is due to the higher viscosity of TBA. This higher viscosity may cover the active sites of the catalyst, resulting in a reduction in catalytic efficiency [58]. When 50 mM p-BQ was added, the degradation efficiency of OFL dropped to 50.5 % (*k*_obs_ = 0.0083 min^−1^), indicating that O_2_^•−^ played a certain role in the degradation process. On the one hand, the formation of O_2_^•−^ may evolve from SO_4_^•−^ and •OH produced by the decomposition of PMS in the system. On the other hand, the dissolved oxygen in the reactive system can also be reduced by electrons to generate O_2_^•−^. Many studies have pointed out that carbon materials generally degrade pollutants through non-radical pathways [59]. Therefore, 3CPC-Mt-p/PMS may have a non-radical reaction pathway to degrade OFL, and ^1^O_2_ is a typical ROS in the non-radical pathway. Therefore, FFA was chosen to quench ^1^O_2_ in the system. When 50 mM FFA was added to the reaction system, only 20.1 % of OFL in the solution was removed (*k*_obs_ = 0.0034 min^−1^), which confirmed that ^1^O_2_ is the main ROS in the 3CPC-Mt-p/PMS reaction system. According to reports, ^1^O_2_ is mainly formed in the following ways: (1) electron transfer occurs between the electron-rich C=O group and PMS, and the SO_5_^•−^ generated by the decomposition of PMS can be converted into ^1^O_2_; (2) •OH generated during the decomposition of PMS can be further converted with O_2_^•−^ in the system to form ^1^O_2_; (3) O_2_^•−^ can also react with H_2_O to form ^1^O_2_ [60,61]. It can be seen from Fig. 5b–d, the order of contribution of reactive oxygen species to OFL degradation is ^1^O_2_ > O_2_^•−^ > •OH.

DMPO and TEMP were used as electron spin capture agents for ESR testing to verify the ROS generated in the 3CPC-Mt-p/PMS reaction system. As shown in Fig. 5b, there is a weak DMPO-•OH characteristic signal peak of 1:2:2:1 in the 3CPC-Mt-p/PMS system, and the DMPO-SO_4_^•−^ characteristic signal peak can be ignored, which shows that there is only a small amount of •OH and almost no SO_4_^•−^ in the system. In addition, to detect the presence of O_2_^•−^ in the system, a large amount of methanol was added to the solution, and then DMPO was added to capture the free radical O_2_^•−^. From Fig. 5c, four characteristic signal peaks corresponding to the DMPO-O_2_^•−^ adduct (relative intensity 1:1:1:1) can be observed. This confirms the generation of superoxide radicals (O_2_^•−^). The 1:1:1 triplet signal in Fig. 5d represents the signal peak of TEMP-^1^O_2_, confirming the existence of ^1^O_2_ in the 3CPC-Mt-p/PMS reaction system. After adding PMS to the solution, a weak TEMP-^1^O_2_ characteristic peak can be observed, which may be attributed to the self-decomposition of PMS in water. However, since the self-decomposition rate of PMS is very slow, the impact on the system is negligible. Notably, when the 3CPC-Mt-p catalyst was added to DMPO/TEMP + PMS, the signal peaks of DMPO-O_2_^•−^ and TEMP-^1^O_2_ were significantly enhanced, which confirmed the high activity of the 3CPC-Mt-p catalyst in the PMS activation process. It also indicates a high concentration of ROS in the system after the addition of the catalyst [62], which is also consistent with the results of free radical quenching experiments.

**Fig. 6:**
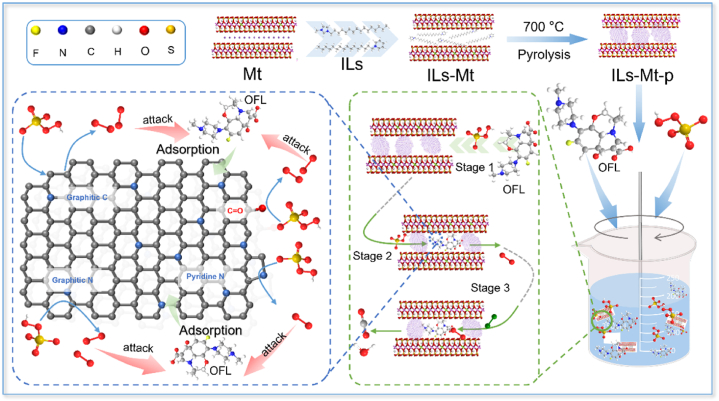
Schematic diagram of the mechanism of 3CPC-Mt-p catalyst-activated PMS to degrade OFL.

In summary, O_2_^•−^ and ^1^O_2_ in the system are the main reactive oxygen species that degrade OFL. The •OH also participates in the reaction, while the contribution of SO_4_^•−^ is negligible. The activation of PMS by 3CPC-Mt-p is mainly mediated by a non-radical pathway, in which ^1^O_2_ is the main active species and O_2_^•−^ and •OH are also involved in the degradation of OFL. Fig. 6 shows a schematic diagram of the mechanism of 3CPC-Mt-p activating PMS to degrade OFL.

### 3.5 Degradation pathways

**Fig. 7:**
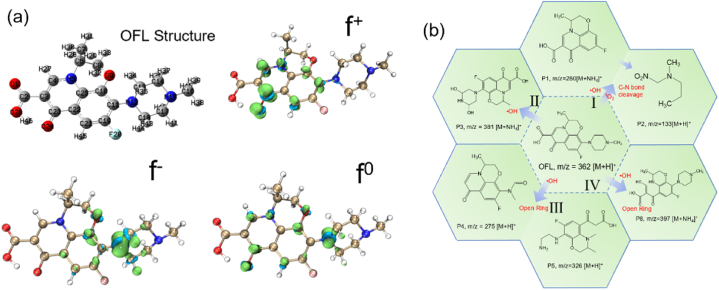
(a) OFL structure with atom labels and Fukui isosurface (ρ = 0.004 a.u.); (b) Schematic diagram of OFL degradation pathway in 3CPC-Mt-p/PMS system.

The pristine OFL (Fig. S10) and various degradation intermediates of OFL in the 3CPC-Mt-p/PMS system (Fig. S11) were determined by liquid chromatography-mass spectrometry (LC-MS). Fukui isosurface derived from DFT calculation was used to predict the possible reaction sites during degradation. The f^−^ isosurface represents the sites easily attacked by the electrophilic reaction, which mainly distribute on C10, C13, C18, N12 and O9 of OFL molecule (Fig. 7a). The f^+^ isosurface can be used to predict the sites for nucleophilic reaction, which located on the C22, C10, C11, C21, N5, and O24. And f^0^ isosurfaces can be used to show the sites for free radical attacks which distribute on C4, C23, C10, C21, N5, N12, O24, O9. Combining the LC-MS intermediates and Fukui results, four OFL degradation pathways were proposed in Fig. 7b. In pathway I, breakage of the C–N bond (C11, N12) between the piperazine ring and benzene resulted in the production of product 1 (P1, *m*/*z* = 279 [M+H]^+^) and product 2 (P2, *m*/*z* = 133 [M+H]^+^). This may be caused by the attack from •OH and ^1^O_2_ [63]. Pathway II ∼ IV can be attributed to the free radical attack of •OH which is consisted of f^0^ results. In pathway II, the piperazine ring (C4, C23) of OFL was attacked by •OH, yielding product 3 (P3, *m*/*z* = 381 [M + NH_4_]^+^). In pathway III, the piperazine ring of OFL is broken (O9), leading to the production of product 4 (P4, *m*/*z* = 275 [M + NH_4_]^+^). The intermediate then continues to be oxidized and the tetrahydropyridine ring is broken, leading to the production of product 5 (P5, *m*/*z* = 326 [M + NH_4_]^+^). In pathway IV, the breakage of the piperazine ring (C4) initiated the degradation of OFL.

### 3.6 Reusability of catalysts

**Fig. 8:**
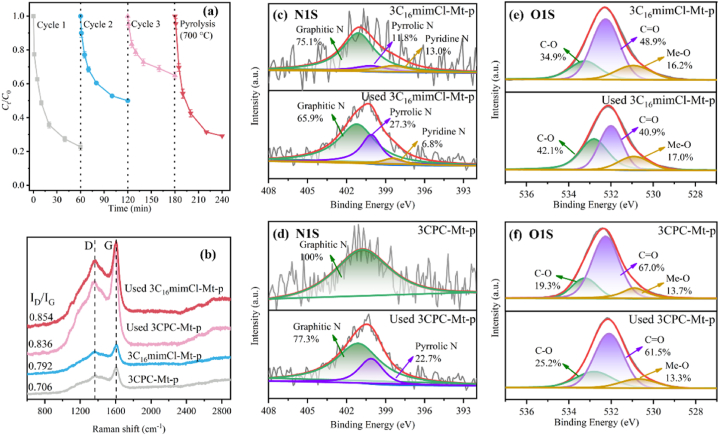
Performance of 3CPC-Mt-p after 3 cycles (a); Raman spectra of 3CPC-Mt-p and 3C_16_mimCl-Mt-p before and after use (b); High-resolution N 1s splitting spectra of 3C_16_mimCl-Mt-p (c) and 3CPC-Mt-p (d); High-resolution O 1s splitting spectra of 3C_16_mimCl-Mt-p (e) and 3CPC-Mt-p (f).

Three cycle stability experiments were conducted to evaluate the reusability and stability of 3CPC-Mt-p to remove OFL in the PMS system. As is shown in Fig. 8a, the decrease in catalyst performance is mainly due to the intermediates formed after OFL decomposition. These intermediates passivate the catalyst surface and cover the active sites. Previous studies have shown that catalyst surface intermediates are not easily removed by washing with common solvents which hinders the functions of active sites and pore structures [64,65]. Therefore, after the third cycle, the catalyst was pyrolyzed for regeneration. The catalytic activity of the calcined and regenerated catalyst almost returned to the level of the fresh catalyst.

To further explore the reasons for the reduced catalytic efficiency, the catalyst materials before and after use were characterized. The mechanism of catalyst deactivation was elucidated through Raman spectroscopy and XPS analysis. As shown in Fig. 8b, after the catalyst was used, the Raman spectrum of the sample still showed typical D-band and G-band. After use, the I_D_/I_G_ values of the 3CPC-Mt-p and 3C_16_mimCl-Mt-p catalysts increased, which indicates that the degree of graphitization of the catalysts was reduced, further confirmed that the graphitized structure plays an important role in the activation of PMS. From Table S6, it can be found that the C and N contents of the catalyst before and after use increased, and the O content decreased, which may be attributed to the degradation intermediates of OFL adsorbed on the catalyst surface. Further, N 1s peak fitting was performed on the catalyst before and after use, as shown in Fig. 8c and d. The result shows that after the catalyst is used, graphite N and pyridine N are converted into pyridine N with worse electron transfer performance. This indicates that the degree of graphitization of the catalyst after use has decreased. This finding is consistent with the conclusion of Raman spectroscopy characterization. Previous studies have shown that a higher degree of graphitization is conducive to the activation of PMS by the catalyst. Therefore, the decrease in the proportion of graphite N may be one of the reasons for the deactivation of the catalyst. According to the O 1s spectrum (Fig. 8e and f), it can be seen that the A_C_=_O_/A_C-O_ of 3CPC-Mt-p and 3C_16_mimCl-Mt-p decreased from 3.47 to 1.40 to 2.44 and 0.97 respectively. Previous studies have shown that C=O groups play a key role in the process of activating PMS to generate ^1^O_2_ [61], so the decrease in the proportion of C=O groups is also one of the possible reasons for catalyst deactivation. The decrease of A_C_=_O_/A_C-O_ further indicates that the degradation intermediates are adsorbed on the surface of the catalyst. This is also a reason for the deactivation of the catalyst.

## 4 Conclusion

In this study, ILs-Mt-p was prepared via the N_2_ high-temperature pyrolysis method and used to activate PMS to degrade OFL. The characterization results show that CPC-Mt-p has a higher degree of graphitization than C_16_mimCl-Mt-p. Graphite N and C=O were observed in the catalyst, which is beneficial to electron transfer. Consequently, it improves the performance to activate PMS. The CPC-Mt-p/PMS system can achieve 77.3 % OFL degradation efficiency within 60 min. The degradation efficiency is negatively correlated with OFL concentration, and satisfactory degradation efficiency is obtained in the pH range of 3.0–9.0. The coexistence of Cl^−^ promotes the degradation of OFL, while both H_2_PO_4_^−^ and HA show a certain inhibitory effect on the degradation of OFL. Through quenching experiments and EPR, it was found that ^1^O_2_ is the main active species. O_2_^•−^ and •OH are also involved in the degradation of OFL. Through Raman and XPS spectra, it was found that graphite N and C=O are important active sites for different organic modification catalysts to generate ^1^O_2_. This study not only provides a montmorillonite composite catalyst for persulfate-based AOPs to treat antibiotic wastewater but also provides new ideas for the construction and regulation of catalyst active sites.

## CRediT authorship contribution statement

**Fu-zhi Huang:** Writing – original draft, Investigation. **Ya-qi Wang:** Writing – review & editing, Investigation. **Wan-yin Gao:** Visualization, Investigation. **Xiao-qiang Cao:** Writing – review & editing, Supervision, Funding acquisition. **Yang Zhang:** Writing – review & editing, Supervision. **Ya-nan Shang:** Writing – review & editing, Supervision. **Yi-zhen Zhang:** Supervision. **Yu-jiao Kan:** Supervision.

## Declaration of competing interest

The authors declare that they have no known competing financial interests or personal relationships that could have appeared to influence the work reported in this paper.
