# Supplementary material for: Construction and regulation of high active sites in montmorillonite composite catalyst for the removal of ofloxacin via persulfate activation
Source: Heliyon. 2024 Apr 22;10(9):e29896. doi: 10.1016/j.heliyon.2024.e29896 (PMC11066642; doi:10.1016/j.heliyon.2024.e29896)
Supplement: Multimedia component 1 [file mmc1.docx]

**Supporting Information**

*for*

**Construction and regulation of high active sites in montmorillonite composite catalyst for the removal of ofloxacin via persulfate activation**

Fu-zhi Huang^1^, Ya-qi, Wang^1^, Wan-yin Gao^1^, Xiao-qiang Cao^1, 2, *^, Yang Zhang^1, 2^, Ya-nan Shang^1, 2, *^, Yi-zhen Zhang^1, 2^, Yu-jiao Kan^1, 2^

*^1^ College of Safety and Environmental Engineering, Shandong University of Science and Technology, Qingdao 266590, China*

*^2^ Institute of Yellow River Delta Earth Surface Processes and Ecological Integrity，Shandong University of Science and Technology, Qingdao 266590, China*

** Corresponding authors: caoxiaoqiang@sdust.edu.cn (X.-q. Cao).*

** Corresponding authors: shangyanan@sdust.edu.cn (Y.-n. Shang).*

# Text S1

**Reagents**

All chemicals, cetylpyridinium chloride (C_21_H_38_ClN, Yancheng Hengyuyuan Chemical Products Co., Ltd., 98%), 1-hexadecyl-3-methylimidazolium chloride (C_20_H_39_ClN_2_, Qingdao Jielong Chemical Co., Ltd., AR), 2-methylimidazole (C₄H₆N₂, Shanghai Chengjie Co., Ltd., AR), hydrogen peroxide (H_2_O_2_, Maclean's Chemical Reagents Ltd., USA, AR), hydrochloric acid (HCl, Jiangsu Xuhong Environmental Protection Technology Co., Ltd., GR), potassium peroxymonosulfate (PMS, Maclean's Chemical Reagents Ltd., USA, 42%~46%), ofloxacin (OFL, Maclean's Chemical Reagents Ltd., USA, 98%), sulfuric acid (H_2_SO_4_, Qingdao Jielong Chemical Co., Ltd., AR), sodium hydroxide (NaOH, Qingdao Jielong Chemical Co., Ltd., AR), sodium chloride (NaCl, Qingdao Jielong Chemical Co., Ltd., AR), anhydrous ethanol (C₂H₅OH, Shandong Jiuchong Chemical Co., Ltd., AR), sodium sulfate (Na_2_SO_4_, Shandong Jiuchong Chemical Co., Ltd., AR), anhydrous sodium carbonate (Na_2_CO_3_, Shandong Jiuchong Chemical Co., Ltd., AR), sodium dihydrogen phosphate (NaH_2_PO_4_, Shandong Jiuchong Chemical Co., Ltd., AR), humic acid (HA, Shandong Jiuchong Chemical Co., Ltd., AR), ethylene glycol (Maclean's Chemical Reagents Ltd., USA, AR), acetonitrile (CH_3_CN ,Tianshengyuan Chemical Co., Ltd., LC), methanol (CH_3_OH, Tixiai Chemical Industrial Development Co., Ltd., LC), tert-butyl alcohol (C₄H_10_O, Tixiai Chemical Industrial Development Co., Ltd., AR), p-benzoquinone (C_6_H_4_O_2_, Yancheng Hengyuyuan Chemical Products Co., Ltd., AR), furfuryl alcohol (C_5_H_6_O_2_, Yancheng Hengyuyuan Chemical Products Co., Ltd., AR), phenol (C_6_H_6_O, Yancheng Hengyuyuan Chemical Products Co., Ltd., AR), 5,5-dimethyl-1-pyrroline-N-oxide (DMPO, Sigma Aldrich Trading Co., Ltd., >97%), 2,2,6,6-tetramethyl-4-piperidone (TEMP, Maclean's Chemical Reagents Ltd., USA, >97%).

# Text S2

**Catalytic Procedure and Mechanism Investigation Experiment**

OFL degradation experiment was conducted in a 150 mL conical flask, proceeding in a constant-temperature oscillator at 140 rpm. The initial pH of the solution was adjusted by NaOH and H_2_SO_4_. At proper sampling time, 0.5 mL of the water samples was filtered with a 0.22 μM filter and 1 mL methanol was added to terminate the degradation reaction. The reactive oxygen species (ROS) generated in the solution were eliminated with corresponding quenchers (Table S1). Different quenchers (methanol (MeOH), tert-butanol (TBA), p-benzoquinone (p-BQ), and furfuryl alcohol (FFA)) were added to quench the reaction and thus determine the type of ROS in the oxidation system. Then used 1 mol/L NaClO_4_ and phenol to capture surface-bound free radicals. The concentration of OFL was determined by a high-performance liquid chromatograph (HPLC) equipped with a C-18 column. All experiments were performed twice in parallel to ensure reproducibility.

The concentration of the target contaminant (OFL) in the solution was determined by HPLC at λ = 294 nm with a mobile phase flow rate of 1.0 mL/min and a column temperature of 25 °C. The mobile phase contained acetonitrile and 0.1% formic acid in a ratio of 15:85. The sample volume was 15 μL, and the retention time of OFL was 6 min.

Pollutant adsorption rate and unit target pollutant adsorption amount as well as pseudo-second-order kinetic fitting model:

| $\text{η}\text{=}\text{（}\text{1}\text{-}\frac{\text{c}}{\text{c}_{\text{0}}}\text{）}\text{×100\%}$ | （1） |
| --- | --- |
| $\text{q}_{\text{e}}\text{=}\frac{\text{c}_{\text{0}}\text{-}\text{c}_{\text{e}}}{\text{W}}\text{×}\text{V}$ | （2） |
| $\frac{\text{t}}{\text{q}_{\text{t}}}\text{=}\frac{\text{1}}{\text{k}\text{q}_{\text{e}}^{\text{2}}}\text{+}\frac{\text{t}}{\text{q}_{\text{e}}}$ | （3） |

In the above formula, *η* represents the adsorption rate of OFL in the solution; *c*_0_ represents the initial concentration of OFL in the solution (mg/L); *c* represents the concentration of OFL in the solution at t min (mg/L); *q*_e_ is the equilibrium adsorption capacity (mg/g); *c*_e_ is the equilibrium concentration of OFL in the solution; *W* is the mass of the adsorbent; *V* is the volume of the solution; *q*_t_ is the adsorption amount at time t (mg/g); *k* is the adsorption pseudo-second-order kinetic reaction rate constant (g/mg·min).

Calculation method of pollutant degradation rate and reaction rate constant (*k*_obs_):

| $\text{ω}\text{ = }\text{（}\text{1-}\frac{\text{c}}{\text{c}_{\text{0}}}\text{）}\text{× 100\%}$ | （4） |
| --- | --- |
| $\text{ln }\text{（}\frac{\text{c}}{\text{c}_{\text{0}}}\text{）}\text{ = -}\text{k}_{\text{obs}}\text{t}$ | （5） |

In the above formula, *ω* represents the degradation rate of OFL in solution, *k*_obs_ is the degradation first-order kinetic reaction rate constant (min^-1^).

# Text S3

**Characterization methods**

X-ray diffractometer (XRD, Rigaku Utima IV, Japan) was used to analyze the crystal structure of the catalyst in the 2θ acquisition range of 1.5-75°, with a scanning speed of 3°/min and an operating voltage of 40 kV, current 40 mA. A Nicolet iS50 Fourier-transform infrared spectrometer (FTIR) from Thermo Fisher, USA, was used to measure the surface functional groups of the catalyst sample in the wave number range of 500-4000 cm^–1^. Scanning electron microscope (SEM) and energy dispersive spectroscopy (EDS) of Apreo S HiVac of FEI, USA, and Talos F200S transmission electron microscope (TEM) of Thermo Fisher were used to observe the surface morphology, particle size and element distribution of different catalyst materials. The specific surface area, pore size and pore volume of the catalysts were determined using a ChemiSorb 2720 BET specific surface area analyzer (BET) from Micromeritics. A variety of gas phase products produced during the pyrolysis process of the catalyst, were measured using the 8860-5977B gas chromatograph-mass spectrometer (GC-MS) of Agilent, USA. GC-MS was equipped with HP-5MS capillary column (30 m×250 μm×0.25 μm), with 1 mL/min high-purity helium as carrier gas. The initial temperature of the column oven was 70 °C, and the temperature was increased to 280 at 20 °C/min °C for 1 min, then raised to 300 °C at 20 °C/min and maintained for 3 min. A Mettler TGA 2 thermogravimetric analyzer (TGA) from Mettler-Toledo Company in Switzerland, was used to analyze the components and thermal stability of the catalysts. A laser Raman spectrometer (LabRAM HR Evolution, Raman) from HORIBA of Japan, was used to analyze the graphitization degree and defect state of the catalysts. The laser spot diameter was 1 μm and the laser frequency was kept below 10 mW. The composition and valence state of the surface of the catalytic material were analyzed using X-ray photoelectron spectroscopy (XPS, ESCALAB250Xi, Thermo Fisher). Electron Paramagnetic Resonance (EPR) experiments were carried out on a JES-FA200 EPR spectrometer to analyze the reactive oxygen species (ROS) involved in the system and the oxygen vacancies on the catalysts surface.

# Text S4

Density functional theory (DFT) calculation was conducted on a Gaussian 09 software [1]. The geometry optimization was performed first according to B3LYP theoretical method on standard 6-31G(d) basis set. Frequency analysis after structural geometry optimization was used to confirm a true energy minimum on the potential energy surface at B3LYP/6-31G(d) level. The single-point energy calculations was obtained at B3LYP/6-311G(d,p) level. Then the Fukui function was analyzed by an electronic wavefunction analysis software of Multiwfn 3.8 [2] and visualized by VMD 1.9.3 software [3]

# Figure


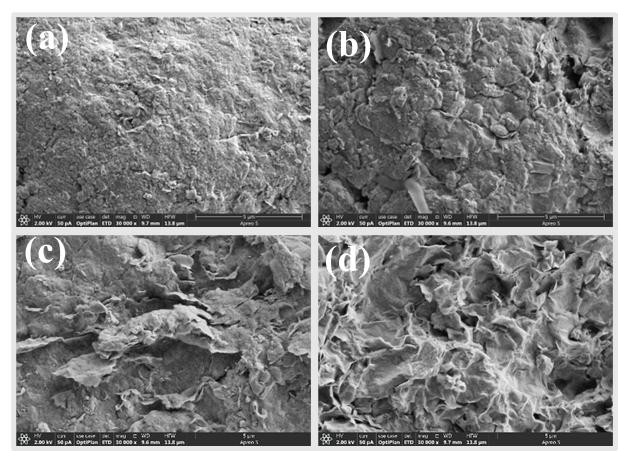


Fig. S1 SEM images: Na-Mt-p (a), 1C_16_mimCl-Mt (b), 3C_16_mimCl-Mt (c) and 3C_16_mimCl-Mt-p (d)

Fig. S2 The quantitative analysis of 3CPC-Mt-p


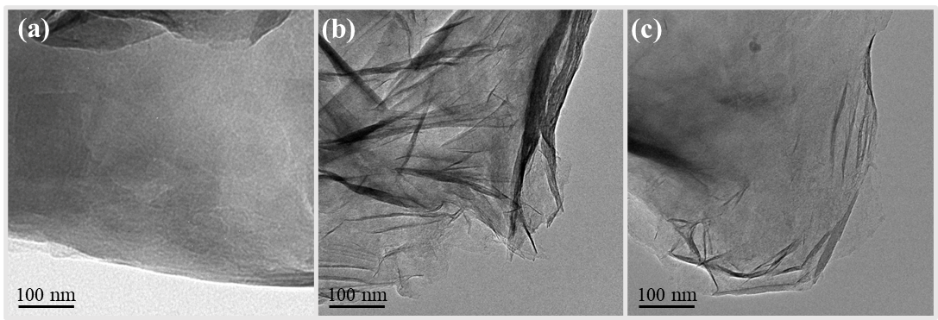


Fig. S3 TEM images: Na-Mt-p (a), 3CPC-Mt-p (b) and 3C_16_mimCl-Mt-p (c)


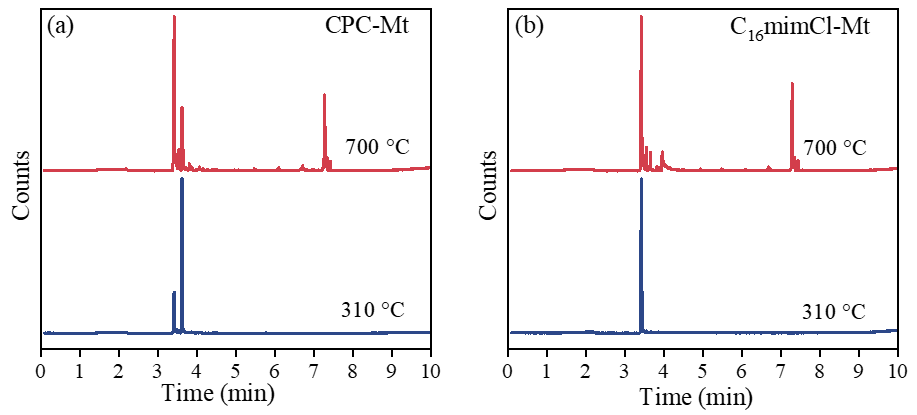


Fig. S4 GC-MS total ion flow (TIC) plots of CPC-Mt (a) and C_16_mimCl-Mt (b)


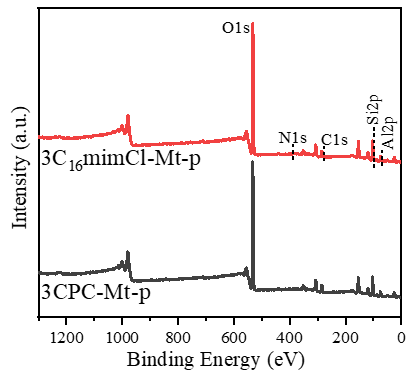


Fig. S5 XPS full spectrum of 3CPC-Mt-p and 3C_16_mimCl-Mt-p

**
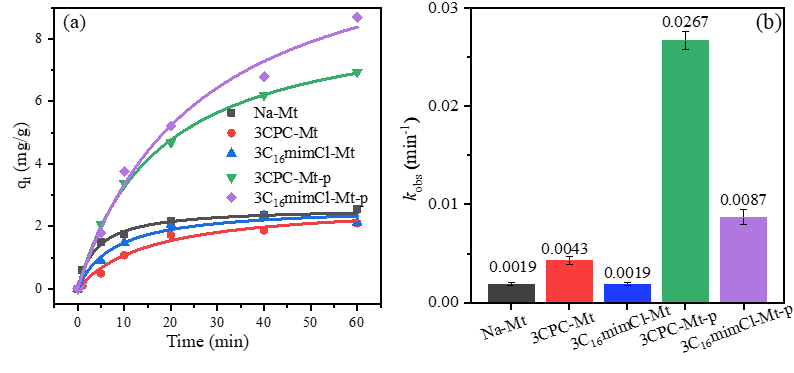
**

Fig. S6 Quasi-secondary kinetic fitting curves for adsorption of OFL by different samples (a)；Degradation reaction rate constant of different samples for OFL(b)


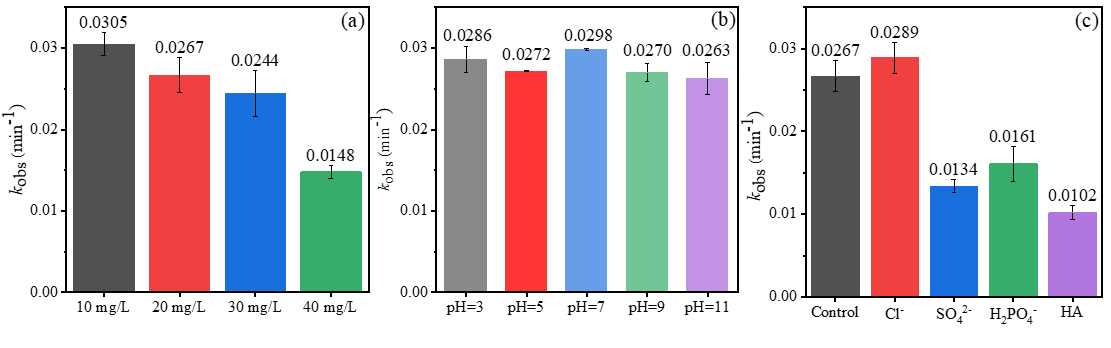


Fig. S7 Effect of pollutant concentration on OFL degradation rate constants (a); Effect of initial pH on OFL degradation rate constants (b); Effect of anions and HA in water on the degradation performance of OFL (c)


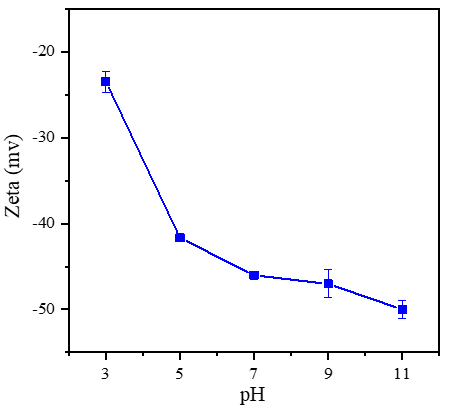


Fig. S8 zeta potential of 3CPC-Mt-p at different pH


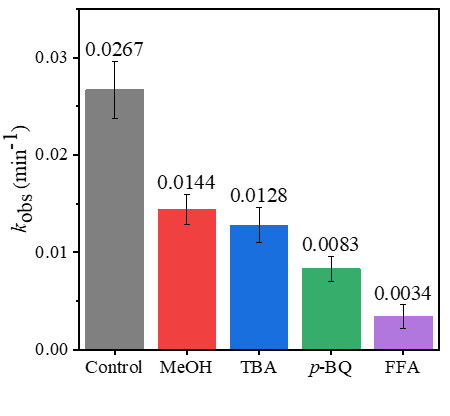


Fig. S9 Effect of quenchers on OFL degradation rate constants

**
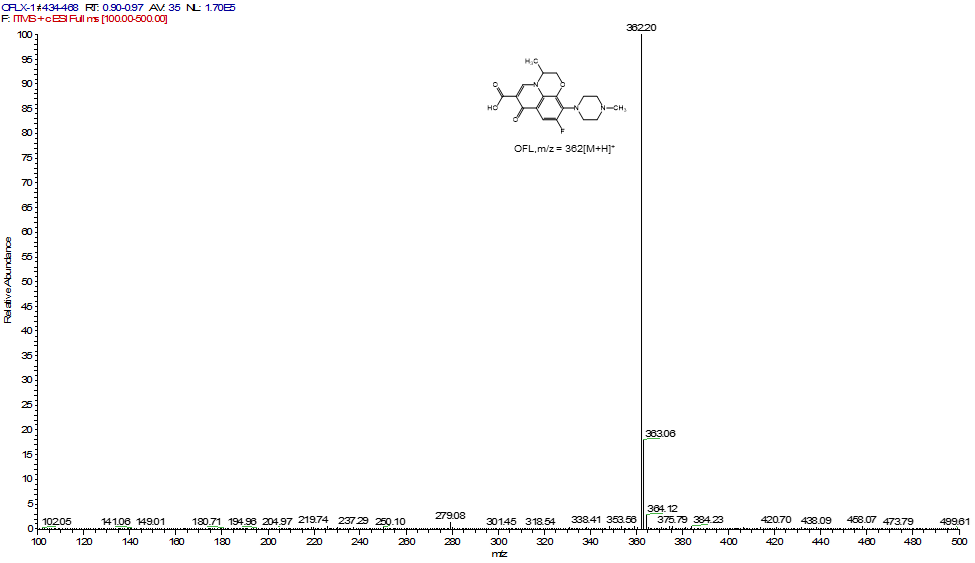
**

Fig. S10. LC-MS spectra of pristine OFL


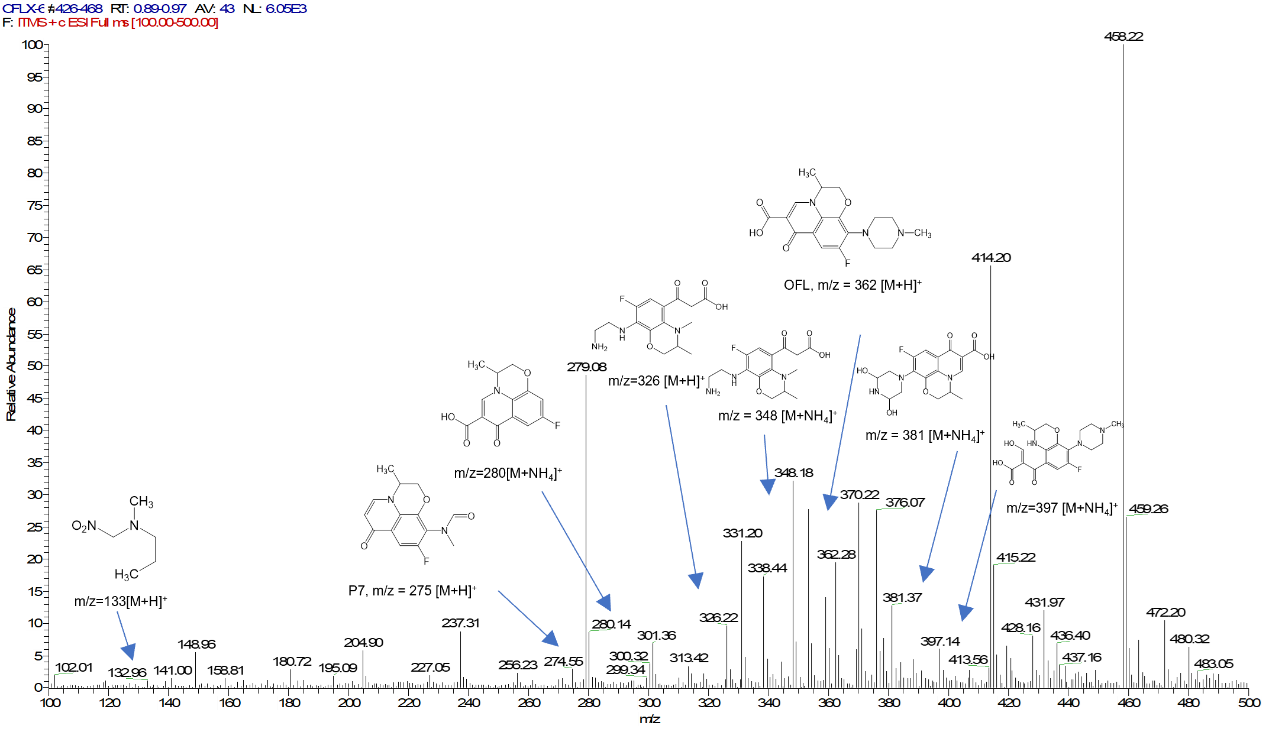


Fig. S11. LC-MS spectra of the intermediates of OFL in the 3CPC-Mt-p/PMS system

# Table

Table S1 Dosage of CPC of nCPC-Mt with 1g montmorillonite

(CEC (montmorillonite) = 82 mmol/100 g, M_C16mimCl_ = 358.01g/mol)

|  | 1CPC-Mt | 2CPC-Mt | 3CPC-Mt | 4CPC-Mt |
| --- | --- | --- | --- | --- |
| Mol of CPC (mol) | 0.82 | 1.64 | 2.64 | 3.28 |
| Mass of CPC (g) | 0.2936 | 0.5871 | 0.8807 | 1.1742 |

Table S2 Dosage of C_16_mimCl of nC_16_mimCl-Mt with 1g montmorillonite

(CEC (montmorillonite) = 82 mmol/100 g, M_C16mimCl_ = 342.99g/mol)

|  | 1C_16_mimCl-Mt | 2C_16_mimCl-Mt | 3C_16_mimCl-Mt | 4C_16_mimCl-Mt |
| --- | --- | --- | --- | --- |
| Mol of C_16_mimCl (mol) | 0.82 | 1.64 | 2.64 | 3.28 |
| Mass of CPC (g) | 0.2812 | 0.5625 | 0.8438 | 1.1250 |

Table S3 Reaction rate constants of different quenchers to ROS

| Quencher | Reaction rate constants *k* of quencher to ROS (M^-1^ s^-1^) | References |
| --- | --- | --- |
| EtOH | *k*_∙OH_ = (1.2-2.8) × 10^9^ M^-1^ s^-1^；*k*_SO4_^•−^ = (1.6–7.7) × 10^7^ M^-1^ s^-1^ | [4] |
| TBA | *k*_∙OH_ = (3.8-7.6) × 10^9^ M^-1^ s^-1^; *k*_SO4_^•−^ = (4-9.1) × 10^5^ M^-1^ s^-1^ | [5] |
| *p*-BQ | *k*_O2_^∙^ = (0.9-1.0) × 10^9^ M^-1^ s^-1^ | [6] |
| FFA | *k*_1O2_ = 1.2×10^8^ M^-1^ s^-1^ | [7] |
| phenol | *k*_phenol+•OH_ = 6.6×10^9^ M^-1^ s^-1^p; *k*_phenol+SO4•−_ = 8.8×10^9^ M^-1^ s^-1^ | [8] |

Table S4 Results of specific surface area and pore structure of different samples

| Sample | Specific surface area  (m^2^/g) | Pore volume  (cm^3^/g) | Average pore diameter  (nm) | |
| --- | --- | --- | --- | --- |
| Na -Mt | 25.8814 | 0.0901 | | 10.0539 |
| 1CPC-Mt | 6.1090 | 0.0430 | | 10.3328 |
| 2CPC-Mt | 1.6399 | 0.0130 | | 14.0252 |
| 3CPC-Mt | 1.2594 | 0.0188 | | 25.2843 |
| 4CPC-Mt | 1.3748 | 0.0201 | | 25.7515 |
| 1C_16_mimCl-Mt | 7.5643 | 0.0445 | | 10.8100 |
| 2C_16_mimCl-Mt | 1.7561 | 0.0126 | | 14.1798 |
| 3C_16_mimCl-Mt | 1.7054 | 0.0233 | | 24.7627 |
| 4C_16_mimCl-Mt | 1.9792 | 0.0115 | | 13.5697 |
| Na-Mt-p | 9.2516 | 0.0800 | | 17.7895 |
| 1CPC-Mt -p | 37.4733 | 0.1351 | | 7.1339 |
| 2CPC-Mt-p | 36.1355 | 0.1402 | | 6.9440 |
| 3CPC-Mt -p | 35.7462 | 0.1891 | | 9.4433 |
| 4CPC-Mt -p | 34.4288 | 0.1698 | | 8.7285 |
| 1C_16_mimCl-Mt -p | 36.0117 | 0.1434 | | 7.4241 |
| 2C_16_mimCl-Mt -p | 33.8777 | 0.1858 | | 8.9360 |
| 3C_16_mimCl-Mt -p | 38.2240 | 0.2176 | | 9.6958 |
| 4C_16_mimCl-Mt -p | 34.6674 | 0.1923 | | 9.5305 |

Table S5 The content ratio of each element of different samples (at.%)

|  | Al | Si | C | N | O |
| --- | --- | --- | --- | --- | --- |
| 1CPC-Mt-p | 9.27 | 21.12 | 8.96 | 0.84 | 59.81 |
| 2CPC-Mt-p | 9.4 | 20.76 | 9.57 | 0.87 | 59.1 |
| 3CPC-Mt-p | 9.11 | 20.8 | 9.96 | 0.96 | 59.18 |
| 4CPC-Mt-p | 9.45 | 21.17 | 8.81 | 0.91 | 59.66 |
| 1C_16_mimCl-Mt-p | 9.48 | 21.15 | 8.61 | 0.82 | 59.93 |
| 2C_16_mimCl-Mt-p | 9.71 | 21.23 | 8.52 | 0.87 | 59.67 |
| 3C_16_mimCl-Mt-p | 9.55 | 21.49 | 8.29 | 0.85 | 59.83 |
| 4C_16_mimCl-Mt-p | 9.5 | 21.66 | 8.22 | 0.84 | 59.77 |

Table S6 Proportion of each element content before and after the use of 3CPC-Mt-p and 3C_16_mimCl-Mt-p (at.%)

|  | Al | Si | C | N | O |
| --- | --- | --- | --- | --- | --- |
| 3CPC-Mt-p | 9.11 | 20.8 | 9.96 | 0.96 | 59.18 |
| 3C_16_mimCl-Mt-p | 9.55 | 21.49 | 8.29 | 0.85 | 59.83 |
| Used 3CPC-Mt-p | 8.57 | 20.41 | 13.37 | 1.65 | 56.00 |
| Used 3C_16_mimCl-Mt-p | 8.81 | 20.29 | 12.72 | 1.81 | 56.37 |

Table S7 The catalytic performance comparison of recently reported Fenton-like catalysts for PMS activation.

| Catalyst | Loading of Catalyst, g L^-1^ | Pollutant (mg L-1) | Removal efficiency | *k*_obs_ | Ref. |
| --- | --- | --- | --- | --- | --- |
| 0.4CF-Mt | 0.4 | OFL (40) | 85.2% | 0.0261 | [9] |
| BC/Mt | 0.5 | TC (20) | 78% | 0.117 | [10] |
| CPANI-9 | 0.025 | Phenol (10μM) | 98% | 0.385 | [11] |
| FMDS-3 | 0.2 | TC (40) | 90.7% | 0.0766 | [12] |
| N-AC/MTN | 0.3 | TC (40) | 86% | 0.35 | [13] |
| Fe_3_O_4_/Mt | 2.0 | DIC (10) | 63% | 0.01 | [14] |
| 10CN-MMT | 0.4 | TC (20) | 80% | 0.0218 | [15] |
| Fe/CPC/TC-Mt-p | 0.6 | OFL (40) | 96.2% | 0.0511 | [16] |
| CuFe_2_O_4_/CuO | 0.5 | CIP (5) | 68.23% | 0.0168 | [17] |
| Fe-MMT | 2.5 | ATZ (5μM) | 94.1% | 0.0361 | [18] |
| CM/Mt | 1.5 | BPA (50) | 98% | / | [19] |
| nZVI/ATP_3_ | 0.5 | QC (20) | 97.36% | / | [20] |
| nCoFe_2_O_4_/OMt | 0.4 | CBZ (5) | 93% | / | [21] |
| 3CPC-Mt-p | 0.4 | OFL (20) | 77.3% | 0.0267 | This Work |

# References

[1] R.A. Gaussian09, 1, mj frisch, gw trucks, hb schlegel, ge scuseria, ma robb, jr cheeseman, g. Scalmani, v. Barone, b. Mennucci, ga petersson et al., gaussian, Inc., Wallingford CT, 121 (2009) 150-166.

[2] T. Lu, F. Chen, Multiwfn: A multifunctional wavefunction analyzer, J. Comput. Chem., 33 (2012) 580-592.

[3] W. Humphrey, A. Dalke, K. Schulten, VMD: visual molecular dynamics, J Mol Graph, 14 (1996) 33-38, 27-38.

[4] X. Chen, W.-D. Oh, T.-T. Lim, Graphene- and CNTs-based carbocatalysts in persulfates activation: Material design and catalytic mechanisms, Chemical Engineering Journal, 354 (2018) 941-976.

[5] Z. Ji, X. Shen, G. Zhu, H. Zhou, A. Yuan, Reduced graphene oxide/nickel nanocomposites: facile synthesis, magnetic and catalytic properties, Journal of Materials Chemistry, 22 (2012) 3471-3477.

[6] W.-D. Oh, Z. Dong, T.-T. Lim, Generation of sulfate radical through heterogeneous catalysis for organic contaminants removal: Current development, challenges and prospects, Applied Catalysis B: Environmental, 194 (2016) 169-201.

[7] H. Xu, W.J. Cooper, J. Jung, W. Song, Photosensitized degradation of amoxicillin in natural organic matter isolate solutions, Water Res., 45 (2011) 632-638.

[8] G. Li, X.-q. Cao, N. Meng, Y.-m. Huang, X.-d. Wang, Y.-y. Gao, X. Li, T.-s. Yang, B.-l. Li, Y.-z. Zhang, X.-j. Lyu, Y. Liang, Fe3O4 supported on water caltrop-derived biochar toward peroxymonosulfate activation for urea degradation: the key role of sulfate radical, Chemical Engineering Journal, 433 (2022) 133595.

[9] X.-q. Cao, F. Xiao, Z.-w. Lyu, X.-y. Xie, Z.-x. Zhang, X. Dong, J.-x. Wang, X.-j. Lyu, Y.-z. Zhang, Y. Liang, CuFe2O4 supported on montmorillonite to activate peroxymonosulfate for efficient ofloxacin degradation, J Water Process Eng, 44 (2021) 102359.

[10] K. Yuan, C. Gao, G. Zhao, H. Yang, Electronic interaction between biochar and montmorillonite toward enhanced peroxymonosulfate activation, Applied Clay Science, 249 (2024) 107260.

[11] S. Liu, Z. Zhang, F. Huang, Y. Liu, L. Feng, J. Jiang, L. Zhang, F. Qi, C. Liu, Carbonized polyaniline activated peroxymonosulfate (PMS) for phenol degradation: Role of PMS adsorption and singlet oxygen generation, Applied Catalysis B-environmental, 286 (2021) 119921.

[12] P. Chen, Z. Cheng, X. Zhang, C. Yan, J. Wei, F. Qiu, Y. Liu, Fe–Mn bimetallic catalyst to activate peroxymonosulfate (PMS) for efficient degradation of tetracycline: Mechanism insights and application for pharmaceutical wastewater, Journal of Cleaner Production, 445 (2024) 141365.

[13] W. Wang, Y. Huang, J. Ni, W. Peng, Y. Cao, Y. Huang, G. Fan, D. Teng, S. Song, In-situ preparation of N-rich nano-activated carbon on negative-charged montmorillonite with enhanced activation of peroxymonosulfate for antibiotics degradation, J Environ Chem Eng, 11 (2023) 111564.

[14] Y. Ouyang, G. Zeng, W. Zhu, X. Yao, M. Yang, X. Long, J. Zheng, Y. Tao, Y. Deng, C. Ding, Highly efficient catalytic degradation of dicamba using Fe3O4/montmorillonite composite: Mechanism and toxicity assessment, Process Safety and Environmental Protection, 176 (2023) 238-248.

[15] M. Li, X. Liu, Z. Xie, C. Du, Y. Su, Anchoring defective metal-free catalysts on montmorillonite nanosheets for tetracycline removal: synergetic adsorption-catalysis and mechanism insights, Environmental Science: Advances, 3 (2024) 290-303.

[16] F. Xiao, Y.-q. Wang, X.-y. Xie, X. Dong, Y. Kan, Y. Zhang, Y.-z. Zhang, G. Zhou, B.-l. Li, X.-q. Cao, J. Zhang, M. Chen, L. Li, X.-j. Lyu, Preparation of Fe/C-Mt Composite Catalyst and Ofloxacin Removal by Peroxymonosulfate Activation, Separation and Purification Technology, (2022).

[17] B. He, L. Song, Z. Zhao, W. Liu, Y. Zhou, J. Shang, X. Cheng, CuFe2O4/CuO magnetic nano-composite activates PMS to remove ciprofloxacin: Ecotoxicity and DFT calculation, Chemical Engineering Journal, 446 (2022) 137183.

[18] P. Wang, X. Liu, W. Qiu, F. Wang, H. Jiang, M. Chen, W. Zhang, J. Ma, Catalytic degradation of micropollutant by peroxymonosulfate activation through Fe(III)/Fe(II) cycle confined in the nanoscale interlayer of Fe(III)-saturated montmorillonite, Water Res., 182 (2020) 116030.

[19] X. Liu, W. Wang, C. Du, Y. Su, Synergistic activation of peroxymonosulfate via oxygen vacancy-rich CoxMn3-xO4/montmorillonite catalyst for environmental remediation, Applied Clay Science, 228 (2022) 106625.

[20] C. Ding, S. Xiao, Y.-C. Lin, P. Yu, M.-e. Zhong, L. Yang, H. Wang, L. Su, C. Liao, Y. Zhou, Y. Deng, D. Gong, Attapulgite-supported nano-Fe0/peroxymonsulfate for quinclorac removal: Performance, mechanism and degradation pathway, Chemical Engineering Journal, (2019).

[21] J. Wu, G. Cagnetta, B. Wang, Y. Cui, S. Deng, Y. Wang, J. Huang, G. Yu, Efficient degradation of carbamazepine by organo-montmorillonite supported nCoFe2O4-activated peroxymonosulfate process, Chemical Engineering Journal, 368 (2019) 824-836.
